# Supplementary material for: Effect of Occupational Stress on Pharmacists’ Job Satisfaction in Saudi Arabia
Source: Healthcare (Basel). 2022 Jul 31;10(8):1441. doi: 10.3390/healthcare10081441 (PMC9408447; doi:10.3390/healthcare10081441)
Supplement: Supplementary file 1 [file healthcare-10-01441-s001.zip › healthcare-1796785-supplementary.pdf]

Supplementary File

Table S1. : Demographics and The Expectation Confirmation Theory Questions

Q1- Please indicate your date of birth:……………..
Q2- Please indicate your gender:<br>Female<br>Male
Q3- Please indicate your monthly salary:………………. Saudi Riyals
Q4- Please indicate your marital status:<br>Single<br>Married<br>Divorced<br>Widow/widower
Q5- If married, how many children?...................Child/ren
Q6- Please indicate your highest Academic qualifications:<br>Bachelor's degree<br>Master's degree<br>Pharm D<br>Completed Residency 1<br>Completed R2<br>Ph.D.
Q7- Please indicate your hours of work per week:…… hours
Q8- Please disclose if you have one of the health problems:<br>No disorder<br>Diabetes<br>Depression<br>Cardiac disorder<br>physical disability<br>Asthma<br>Other, please write it down……………….
Q9- Please indicate your work experience (Year):………………. Years
Q10- How many non-morning shifts are assigned to you per month?<br>One time per month<br>Two-three times per month<br>Four- five times per month<br>More than five times per month
Q11-Please indicate the type of pharmacy job?:<br>Community pharmacist<br>Hospital pharmacist<br>Consultant of pharmacy<br>Pharmaceutical company<br>Faculty by educational institute<br>Saudi Food and Drug Authority<br>Insurance company<br>Other, please write it down……………….
Q-12 - Please indicate who is responsible for paying your wage?<br>Governmental<br>Private<br>Military
Q13- Please indicate the average number of beds:…………………bed
Q14-Please answer the following questions regarding the level of satisfaction in your job as a pharmacist:<br>I am ______ with my work.

Extremely displeased/Extremely pleased
Extremely frustrated/Extremely contented
Extremely miserable/Extremely delighted
Extremely dissatisfied/Extremely satisfied

Q15- Please answer the following questions regarding your level of expectation before starting your job as a pharmacist:
Before I practiced the profession of pharmacy, I expected to receive a rewarding salary.
Before I practiced the profession of pharmacy, I expected to get a stable job.
Before I practiced the profession of pharmacy, I expected to get a high position.
Before I practiced the profession of pharmacy, I expected to get more educational opportunities.
Before I practiced the profession of pharmacy, I expected to get more promotion opportunities.

Q16- Please answer the following questions regarding your job performance after starting your job as a pharmacist:
After practicing the profession of pharmacy, I was able to get a rewarding salary.
After practicing the profession of pharmacy, I was able to get a stable job.
After practicing the profession of pharmacy, I was able to obtain a high position.
After practicing the profession of pharmacy, I was able to get more educational opportunities.
After practicing the profession of pharmacy, I was able to get more promotion opportunities.

Table S2. ERI Questionnaire short version

		Strongly Disagree	*Disagree*	*Agree*	*Strongly Agree*
**ERI1**	I have constant time pressure due to a heavy work load.	□	□	□	□
**ERI2**	I have many interruptions and disturbances while performing my job.	□	□	□	□
**ERI3**	I have a lot of responsibility in my job.	□	□	□	□
**ERI4**	I am often pressured to work overtime.	□	□	□	□
**ERI5**	My job is physically demanding.	□	□	□	□
**ERI6**	Over the past few years, my job has become more and more demanding.	□	□	□	□
**ERI7**	I receive the respect I deserve from my superior or a respective relevant person.	□	□	□	□
**ERI8**	I experience adequate support in difficult situations.	□	□	□	□
**ERI9**	I am treated unfairly at work. Reverse coding	□	□	□	□
**ERI10**	My job promotion prospects are poor. Reverse coding	□	□	□	□
**ERI11**	I have experienced or I expect to experience an undesirable change in my work situation. Reverse coding	□	□	□	□
**ERI12**	My employment security is poor. Reverse coding	□	□	□	□
**ERI13**	My current occupational position adequately reflects my education and training.	□	□	□	□
**ERI14**	Considering all my efforts and achievements, I receive the respect and prestige I deserve at work.	□	□	□	□
**ERI15**	Considering all my efforts and achievements, my job promotion prospects are adequate.	□	□	□	□
**ERI16**	Considering all my efforts and achievements, my salary / income is adequate.	□	□	□	□
